# Retrospective Analysis of Emotional Burden and the Need for Support of Patients and Their Informal Caregivers after Palliative Radiation Treatment for Brain Metastases

**DOI:** 10.3390/curroncol29060338

**Published:** 2022-06-11

**Authors:** Jamie Lütscher, Christa Hauswirth Siegenthaler, Caroline Hertler, David Blum, Paul Windisch, Renate Grathwohl Shaker, Christina Schröder, Daniel Rudolf Zwahlen

**Affiliations:** 1 Institute for Radiation Oncology, Cantonal Hospital Winterthur, 8401 Winterthur, Switzerland; jamie.luetscher@ksw.ch (J.L.); paul.windisch@ksw.ch (P.W.); christina.schroeder@ksw.ch (C.S.); 2 Center for Palliative Care, Cantonal Hospital Winterthur, 8401 Winterthur, Switzerland; christa.hauswirth@ksw.ch (C.H.S.); renate.grathwohlshaker@ksw.ch (R.G.S.); 3 Department of Radiation Oncology, Competence Center for Palliative Care, University Hospital and Zurich, 8091 Zurich, Switzerland; caroline.hertler@usz.ch (C.H.); david.blum@usz.ch (D.B.)

**Keywords:** informal caregivers, needs for support, emotional burden, palliative care, brain metastases

## Abstract

Cancer burdens not only the patients themselves but also their personal environment. A few studies have already focused on the mental health and personal needs of caregivers of patients. The purpose of this retrospective analysis was to further assess the emotional burden and unmet needs for support of caregivers in a population of brain metastasis patients. In the time period 2013–2020, we identified 42 informal caregivers of their respective patients after palliative radiation treatment for brain metastases. The caregivers completed two standardized questionnaires about different treatment aspects, their emotional burden, and unmet needs for support. Involvement of psycho-oncology and palliative care was examined in a chart review. The majority of the caregivers (71.4%, *n* = 30) suffered from high emotional burden during cancer treatment of their relatives and showed unmet needs for emotional and psychosocial support, mostly referring to information needs and the involvement in the patient’s treatment decisions. Other unmet needs referred to handling personal needs and fears of dealing with the sick cancer patient in terms of practical care tasks and appropriate communication. Palliative care was involved in 30 cases and psycho-oncology in 12 cases. There is a high need for emotional and psychosocial support in informal caregivers of cancer patients. There might still be room for an improvement of psychosocial and psycho-oncological support. Care planning should cater to the emotional burden and unmet needs of informal caregivers as well. Further prospective studies in larger samples should be performed in order to confirm this analysis.

## 1. Introduction

Cancer patients have been in the focus of care and support from both their oncologic caregivers and their psycho-oncologist or palliative care specialists for a long time [1]. However, there is still a need to improve the focus on their family or informal caregivers since cancer influences and burdens the entire personal environment of the patients [1,2,3,4].

Informal caregivers are defined as the most important reference persons for the patient. The emotional burden and the personal needs of these informal caregivers are often insufficiently addressed [5,6,7,8] although the informal caregiver is known to suffer as well and fill the role of the key supporter of the patient [1,2]. The strain of being the patient’s supporter combined with general concerns for the patient pose a risk of developing mental and physical health problems [1,2]. According to a study by Pitceathly et al. [2], 30–50% of the informal caregivers suffer from psychological distress such as depression, anxiety, or adaption disorders. This psychological pressure increases with the demand for care and the dependency of the patient, for example, in patients with neurological deficits or personality changes, as seen in patients with tumors located in highly functional brain regions often caused by primary or secondary brain tumors [1].

Primary brain tumors are tumors developed from the brain parenchyma and its surrounding structures and show an incidence of approximately 11:100,000 person years [9]. Secondary brain tumors develop when tumor cells from a distant primary tumor, most frequently lung, breast, or colorectal cancers as well as melanoma and renal cell carcinoma, metastasize to the brain [10]. The incidence of brain metastases is heterogeneously described; the numbers vary based on the primary tumor type and might be increasingly diagnosed due to improved diagnostic methods, with estimates of an incidence of 7–14:100,000 person years [11].

Typical brain metastases-related symptoms such as neurocognitive impairment, headaches, severe fatigue, dysphagia, personality changes, fluctuating vigilance, epileptic seizures, and loss of mobility create a high dependency on the caregivers [8,12,13,14]. The cerebral radiation effects and chemotherapy induced side effects as well as the short life expectancy of the patients intensify the difficulty for the caregivers of both patients with brain metastases and primary brain tumors [13]. Nevertheless, caregivers of patients with brain metastases might suffer from a different burden than caregivers of patients with primary brain tumors as patients with brain metastases have in physically and mentally managing not only the consequences of the brain tumor itself but also of its distant primary tumor. Thus, patients with brain metastases show a high morbidity and mortality with negative influence on the quality of life of both the patients and the caregivers [14]. As the caregivers’ own mental health is often seen as trivial in comparison to the patient’s suffering, the caregivers start repressing their own needs and desires in order to support the patient [1]. This increased use of support by the patients could therefore lead to a need for support by the caregivers themselves in order to remain mentally healthy and stay able to care for the patient [1,15].

This opens up the question of which of the informal caregivers’ needs to remain unnoticed during cancer treatment of patients and how support for the caregivers could be improved. Therefore, we conducted a retrospective study to assess the emotional burden and unmet emotional and psychosocial needs for support of informal caregivers of patients who received palliative radiotherapy for brain metastases. We hypothesized that there were still some unmet needs of the informal caregivers and that the identification of unmet needs could help to improve means of support and thereby ease the emotional burden of the caregivers in the future.

## 2. Materials and Methods

This analysis is a retrospective study based on a questionnaire survey addressing the informal caregivers of patients treated for brain metastases.

We identified caregivers of deceased patients after palliative radiation treatment for brain metastases at our department of Radiation Oncology at the Winterthur Cantonal Hospital between 2013 and 2020. Patients and caregivers had to be ≥18 years of age and proficient in German. All patients had suffered from solid tumors presenting with brain metastases and had shown an Eastern Cooperative Oncology Group (ECOG) Performance status 0–2 at first presentation for brain radiotherapy. Some of the patients received palliative care by a palliative care specialist for advanced care planning or psychosocial support, usually after treatment goals were changed to best supportive care or end-of-life care. The palliative care service at the Cantonal Hospital of Winterthur, as part of the department of internal medicine, works in close collaboration with the department of medical oncology. The systemic oncologic therapy and radiation therapy was delivered in the same hospital but in different facilities with close cooperation.

The caregivers were contacted by telephone and received information material as well as two questionnaires by mail. The first non-validated and self-designed questionnaire contained 15 open and closed questions about the emotional burden of the caregivers measured on a five point subjective scale, the sufficiency of information the caregivers received, as well as the care and development of health problems the caregivers experienced during their relatives’ disease. The questionnaire also asked about treatment options, the place of death, and the involvement of palliative care. The second validated and standardized questionnaire, the Supportive Care Needs Survey for Partners and Caregivers (SCNS P&C-G), assessed the emotional and psychosocial needs for support of caregivers and consisted of 45 multiple choice questions about the caregivers’ satisfaction of information procurement, the involvement of medical care, nursing and treatment of the patients, as well as the internal medical coordination system. The caregivers’ own needs, health status, and changes in lifestyle were also considered, including fears and concerns. Furthermore, the questionnaire took the change of the relationship and communication method between the caregiver and the patient into account.

The collection of data was approved by the local Ethics Committee (reference number/BASEC ID 2020-02124). The approval of the survey does not lie within the formal scope of responsibility of the local ethics committee, but the study was acknowledged by the Ethics Committee. Informed consent for the questionnaire survey was obtained from the caregivers. Anonymity was guaranteed by encoding the questionnaires.

The statistical analysis was performed using the statistical software “Statistical Package for the Social Science” (SPSS) of IBM (version 25; International Business Machines Corporation IBM, Armonk, NY, USA). For group comparison, Mann–Whitney U test was used. The level of significance was defined as α = 0.05 (5%).

## 3. Results

### 3.1. Patient and Caregiver Characteristics

**Figure A1:**
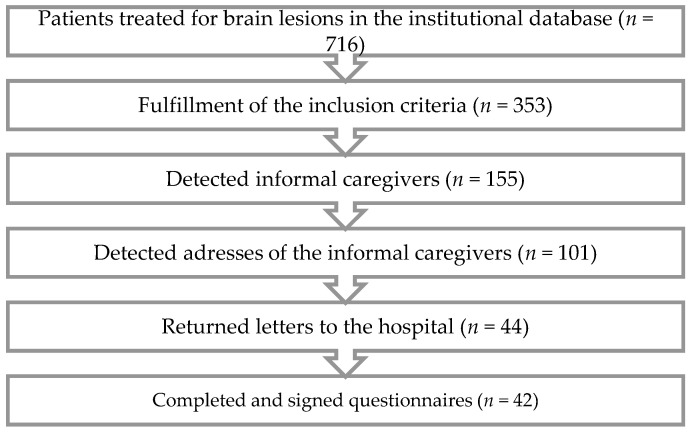
Selection process of the study population.

A total of 716 patients treated for brain lesions were identified in the institutional database. In all, 353 patients fulfilled the inclusion criteria mentioned above. We could trace 155 informal caregivers, of which 54 had to be excluded due to undocumented changes in address. Of the 101 letters that were sent to the caregivers, 44 letters were returned with completed questionnaires (response rate 43.6%). Two letters had to be excluded due to a missing signature and unmet inclusion criteria. In total, 42 informal caregivers were included in this analysis. This selection process is also illustrated in Appendix A (Figure A1).

The majority of the deceased patients suffered from lung cancer. They showed a median number of 4 organ metastases and >10 brain metastases on average, which were treated with whole brain radiotherapy (WBRT) in 86.1% (*n* = 37). Only a small number of patients underwent partial brain radiotherapy (PBRT, 4.7%, *n* = 2) or stereotactic radiosurgery or therapy (SRS/SRT, 9.3%, *n* = 4). Patients received end-of-life care in the hospital rather than at home.

The caregivers considered in this study were all relatives of the patients. The majority were spouses (80.9%, *n* = 34), followed by daughters (9.5%, *n* = 4) and sisters (9.5%, *n* = 4). Most of the caregivers lost their relatives in the year 2019 (23.8%, *n* = 10) at an average age at death of 68 years. The majority of caregivers were female (64.3%, *n* = 27) for an equal number of male and female patients (female 50%, *n* = 21; male 50%, *n* = 21).

Further information about patient and caregiver characteristics can be found in Table 1 and Table 2.

### 3.2. The Caregivers’ Burden and Support

Overall, 78.6% (*n* = 33) of the informal caregivers suffered from high emotional burden during cancer treatment of their relatives. Another 23.8% (*n* = 10) developed health problems, such as back pain, exhaustion, weight gain, sleeping disorders, and mental problems. The majority of the caregivers felt sufficiently supported by their family (88.1%, *n* = 37) as well as by medical practitioners (83.3%, *n* = 35). In total, palliative care was involved in 71.4% (*n* = 30), whereas only 28.6% (*n* = 12) were supported by psycho-oncological care (Table 3).

### 3.3. The Caregivers’ Unmet Needs

According to the caregivers’ needs, the highest needs for support were recorded when dealing with fears about physical or mental deterioration of the patient (50.0%, *n* = 21), when reducing stress of the patient (38.1%, *n* = 16), or when receiving emotional support for themselves (35.7%, *n* = 15). Handling thoughts about death or dying was also a frequent cause of needing support (35.7%, *n* = 15). Other needs, such as the need for support when communicating with the patient (31.0%, *n* = 13); respecting the caregiver’s health, including eating and sleeping (31.0%, *n* = 13); or balancing the needs of the patient and the caregiver (33.3%, *n* = 14), were also reported as relevant but from a smaller quantity of caregivers. Furthermore, nursing affected in 26.1% (*n* = 11) of the cases the caregiver’s own life. Support in practical care tasks, such as bathing, bandage changes, or administering medicine, was applied for in fewer times than other examination points in this study (28.6%, *n* = 12) (Table 4). Due to personal reasons, a small number of caregivers chose to skip individual questions concerning unmet needs of support and hospital coordination. We illustrated those missing values in Table 4 and Table 5.

### 3.4. Health Care Service and Information Needs

Concerning the health care service and information needs, a large number of caregivers underlined the need for support when receiving opportunities to discuss their concerns with doctors (47.6%, *n* = 20). Additionally, many caregivers lacked support when building confidence in doctors having discussed the patient’s case sufficiently (45.2%, *n* = 19) or when receiving information about the supportive program for the caregiver (45.2%, *n* = 19). Furthermore, 42.9% (*n* = 18) of the caregivers pointed out a need for support when being certain about sufficient coordination of medical services, whereas only a small number of caregivers underlined the need for support when participating in treatment decisions (33.3%, *n* = 14) or medical care (26.2%, *n* = 11) of the patient (Table 5).

### 3.5. Palliative Care

**Figure A2:**
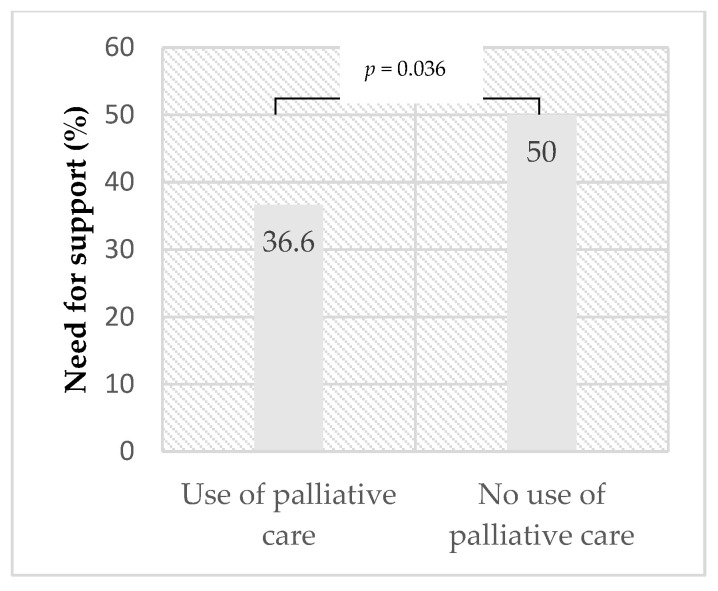
Sufficient case conference and the use of palliative care: confidence in doctors’ decisions with and without the use of palliative care, with data in percentage (%), Mann–Whitney U test, *p* < 0.05.

Palliative care was involved in treatment plans of 30 patients (71.4%). Only 12 patients (28.6%) did not receive palliative care consultation (Table 3). A significant difference in the involvement of palliative care could be observed when feeling confident in good cooperation of doctors: 50.0% (*n* = 6) of caregivers of patients who did not receive palliative care expressed their need for more support, whereas only 36.6% (*n* = 11) of caregivers of patients who received palliative care showed a further need for support. This difference is also illustrated in Appendix A (Figure A2).

## 4. Discussion

Although informal caregivers are the key supporters of patients and have to withstand high emotional burden, they receive little attention and support in the oncological therapy setting [1,2,7]. This study examines the emotional burden of informal caregivers of patients treated for brain metastases and explores unmet needs of informal caregivers in such situations.

In our analysis, the majority of the informal caregivers suffered from high emotional burden during the cancer disease of their relatives. According to Kraehenbuehl et al. [16] and Soothill et al. [7], the emotional distress of informal caregivers might even be more pronounced than the psychological burden of the patients themselves. Here, nearly one-quarter of all informal caregivers developed health problems, such as back pain, exhaustion, weight gain, sleeping disorders, and mental problems, associated with the patient’s disease and care. These physical and mental health problems in caregivers may potentially result in a reduced capacity to support the patient, which may lead to worse outcomes [1]. Likewise, it has been shown in previous studies that patients with low social support generally show higher disease progressions and lower survival times than patients with high social support [17]. Therefore, it is very important to detect and ease the emotional burden of the caregivers.

In our analysis, we identified several unmet needs of caregivers. The majority of the caregivers reported emotional and psychosocial needs for support, which is in line with several other studies [7,8,18]. These unmet needs were highly individual and varied among caregivers. Pursuant to a study of Soothill et al. [7], caregivers with unmet needs often lacked social support, suffered from poor health conditions themselves, or were challenged with multiple caring responsibilities at the same time. Regarding gender differences, Flechl et al. stated that female caregivers felt more often insufficiently informed by medical practitioners than male caregivers [19], which is an important observation since care work tends to be carried out more often by female caregivers [8,19,20]. The fact that caregivers are predominantly female could also be confirmed in our study: the majority of the informal caregivers in this analysis were female, whereas the patients they cared for were equally often male and female (50% male, 50% female). Traditional gender roles but also the fact that women tend to participate in surveys more often than men might have contributed to that difference [19].

In this analysis, we could identify needs for support of the caregivers concerning social interactions with the patient, for example, when communicating with the patient and when reducing the emotional burden of the patient. The reason might be that many caregivers avoid talking about the disease course in the presence of their relatives suffering from cancer due to the fear of depriving them of hope [1]. Assistance in how to talk and deal with terminally ill people could potentially be beneficial regarding this aspect.

Additionally, this analysis could identify needs for support concerning the caregivers’ own health condition. The caregivers wish for support when balancing their own needs and the needs of the patients and when looking after their own health. This is likely because the existential threat of the cancer disease of their relatives makes caregivers neglect their own needs and health problems [1]. Thus, support programs for caregivers should be organized in a way that allows them to both take care of themselves as well as of their ill relatives [7].

Another unmet need was the fear concerning the health deterioration or death of the patient. In these cases, an open communication method might also be referred to, whereby communication should be facilitated by joint discussions of both the patient and the caregiver [1]. Discussions about the patient’s preferred place of care and of death as well as treatment plans with instructions for the control of burdensome symptoms during end-of-life care are crucial to ensure a better end-of-life care and to prevent undesired rehospitalizations [8].

Furthermore, our study stated that providing informal care is burdensome and affects the caregiver’s own life significantly [14]. The emotional burden might even increase in the future since in-patient care will be transferred more and more into outpatient settings, partially due to new treatment possibilities or economic re-structuration of health care [15,21].

As our study shows, there is still room for improvement in the communication with caregivers. For example, caregivers wish to be better informed and more involved in decision making as well as in the medical care of their relatives. This observation is in accordance with several other studies, which underline the importance of an active involvement of caregivers in treatment plans, in decision making, and the reception of information about the health status of the patient [1,7,14,22]. According to the caregivers’ opinion, they experience a lack of opportunities to discuss their concerns and questions with doctors. This might also explain the caregivers’ uncertainty concerning sufficient coordination and implementation of medical services detected in our study. Therefore, we suggest addressing the caregivers needs for specific support openly at the very beginning of any palliative treatment.

Specialized palliative care, either in the in-patient or out-patient setting, was involved in the majority of the patients (71.4%, *n* = 30). However, the inclusion of psycho-oncological care could only be identified in about 28.6% (*n* = 12) of the patients. Expanding the involvement of psycho-oncological care as an offering to both the caregiver and the patient might enhance the multi-professional support, including psychosocial and psycho-oncological care, and consequently empower patients and caregivers to better cope with the emotional stress and the disease of the patient [1,23]. Several previously published studies indicated that advanced care planning affords the informal caregivers a reduction of the emotional burden and a more self-determined remaining lifetime after the patient’s death [1,20]. Although palliative interventions were not aligned explicitly with the informal caregivers but with the patients themselves, the confidence of caregivers in a good collaboration with doctors was higher in those whose relatives had received palliative care consultation. This might represent a positive association between the involvement of palliative care and the confidence in medical practitioners. Hereafter, care planning should more often cater to the emotional burden und unmet needs of the caregivers as well. Nevertheless, the small number of cases and the fact that we conducted a retrospective study does not confirm a causality. A follow-up prospective study would be necessary to confirm these observations.

A possible limitation of this study might lie in the fact that only a small sample of informal caregivers took part in our questionnaires, which allows only for limited interpretation. Nevertheless, our response rate of 43.6% reaches the expected range of studies involving caregivers of deceased patients and is comparable to other studies [16,24]. Additionally, the caregivers were not involved in the design of the non-validated questionnaire, so the questionnaires tend to represent the clinicians’ opinion of which needs seem to be important rather than the opinion of the person with the lived experience. Although no repeated comments in the open question at the end of the non-validated questionnaire could be found, a caregiver representative would have been an important advisor to design the individual questionnaire. Furthermore, the heterogenic cohort of both patients and caregivers might constitute another limitation of this study though covering a broad collective with well-balanced sex of the patients. With only German-speaking caregivers, no conclusion about cultural or ethnical differences could be made. Additionally, the analysis dates back over seven years, so it is very likely that at least some caregivers might be affected by some recall bias. Moreover, the study did not differentiate between the caregiver’s burden experience between the primary tumor and brain metastases. Any symptom load triggered by the primary tumor outside of the brain might influence the subjective burden beyond the brain metastases. Finally, no further differentiation between the different treatment methods of palliative care was made. Therefore, further prospective studies should be performed in order to limit these biases, including a caregiver representative in the process of the study set-up.

## 5. Conclusions

In conclusion, our study shows that informal caregivers of brain metastasis patients suffer from high emotional burden and show a high need for support. Whereas the majority of patients treated for brain metastasis at our institution received some form of palliative care support during the treatment course, there is still room for an improvement of the psychosocial and psycho-oncological support for informal caregivers. Care planning should focus on the emotional burden and unmet needs of informal caregivers with implementation of emotional support systems. Further prospective studies in larger samples should be performed in order to confirm the benefit of well-structured multi-professional palliative care programs for patients with brain metastasis and their informal caregivers.

## Figures and Tables

**Table 1:** Patients’ characteristics at the time of first diagnosis of brain metastases (*n* = 42).

Variables	Median	Range
Age of death (years)	68	47–85
Number of organ metastases	3.9	1–8
ECOG performance status at start of RT	1.4	0–2
Disease duration (first diagnosis to death), (years)	3.1	0–19
	***n***	**%**
Sex		
Female	21	50
Male	21	50
Form of overall treatment (any treatment time)		
Chemotherapy	30	71.4
Immunotherapy	4	9.5
Targeted Therapy	17	40.5
Hormone Therapy	8	19
Radiotherapy in total	42	100
WBRT	37	86.1
PBRT	2	4.7
SRS/SRT	4	9.3
Surgery	17	40.5
Entity		
Lung cancer	26	61.9
Breast cancer	4	9.5
Melanoma	4	9.5
Urogenital cancer	5	11.9
Others	3	7.1
Number of brain metastases		
1–5 metastases	9	21.4
6–10 metastases	6	14.3
>10 metastases	20	47.6
Meningeal carcinomatosis	7	16.7
Place of death		
Home	13	31
Hospital	20	47.6
Nursing home	8	19
Hospice	1	2.4

**Table 2:** The caregiver’s characteristics (*n* = 42).

Variables	*n*	%
Sex		
Female	27	64.3
Male	15	35.7
Relatives		
Spouse	34	81.0
Daughter	4	9.5
Sister	4	9.5

**Table 3:** The caregiver’s burden and support, with data in total number (*n*) and percentage (%) for categorical variables.

Variables	*n* = 42	%
Emotional burden of the caregiver	33	78.6
Sufficient support by medical practitioners	35	83.3
Sufficient support by family	37	88.1
Health problems developed by caregiver	10	23.8
Involvement of palliative care	30	71.4
Outpatient	16	38.1
Inpatient	4	9.5
Unspecified	10	23.8
Psycho-oncology involved	12	28.6

**Table 4:** The caregiver’s unmet needs, with data in total number (*n*) and percentage (%) for categorical variables.

Need for Support When	No Need	Already Supported	Low Need	Moderate Need	High Need	Missing
Receiving emotional support of the caregiver	10 (23.8)	8 (19.0)	7 (16.7)	4 (9.5)	4 (9.5)	9 (21.4)
Coping with fears about physical or mental deterioration of the patient	7 (16.7)	8 (19.0)	7 (16.7)	5 (11.9)	9 (21.4)	6 (14.3)
Handling thoughts about death or dying	9 (21.4)	13 (31.0)	8 (19.0)	1 (2.4)	6 (14.3)	5 (11.9)
Providing practical care tasks (bathing, bandage changes, administering medicine)	16 (38.1)	7 (16.7)	6 (14.3)	3 (7.1)	3 (7.1)	7 (16.7)
Communicating with the patient	15 (35.7)	4 (9.5)	5 (11.9)	2 (4.8)	6 (14.3)	10 (23.8)
Reducing stress of the patient	8 (19.0)	12 (28.6)	5 (11.9)	3 (7.1)	8 (19.0)	6 (14.3)
Balancing the needs of the patient vs. those of the caregiver	12 (28.6)	7 (16.7)	6 (14.3)	5 (11.9)	3 (7.1)	9 (21.4)
Looking after the caregiver’s health (eating, sleeping)	14 (33.3)	10 (23.8)	5 (11.9)	5 (11.9)	3 (7.1)	5 (11.9)
Nursing affects the caregiver’s own life	13 (31.0)	6 (14.3)	5 (11.9)	3 (7.1)	3 (7.1)	12 (28.6)

**Table 5:** Hospital coordination, with data in total number (*n*) and percentage (%) for categorical variables.

Need for Support When	No Need	Already Supported	Low Need	Moderate Need	High Need	Missing
Receiving opportunities to discuss the caregivers concerns with the doctors	9 (21.4)	8 (19.0)	5 (11.9)	8 (19.0)	7 (16.7)	5 (11.9)
Building confidence in doctors having discussed the patient’s case sufficiently with each other	10 (23.8)	7 (16.7)	5 (11.9)	5 (11.9)	9 (21.4)	6 (14.3)
Feeling reassured about sufficient coordination of medical services	10 (23.8)	7 (16.7)	6 (14.3)	2 (4.8)	10 (23.8)	7 (16.7)
Participating in decision making of the patient	11 (26.2)	9 (21.4)	6 (14.3)	4 (9.5)	4 (9.5)	8 (19.0)
Being involved in the medical care of the patient	8 (19.0)	13 (31.0)	3 (7.1)	4 (9.5)	4 (9.5)	10 (23.8)
Receiving information about the supportive program for the caregiver	10 (23.8)	8 (19.0)	6 (14.3)	6 (14.3)	7 (16.7)	5 (11.9)

## Data Availability

Not applicable.
